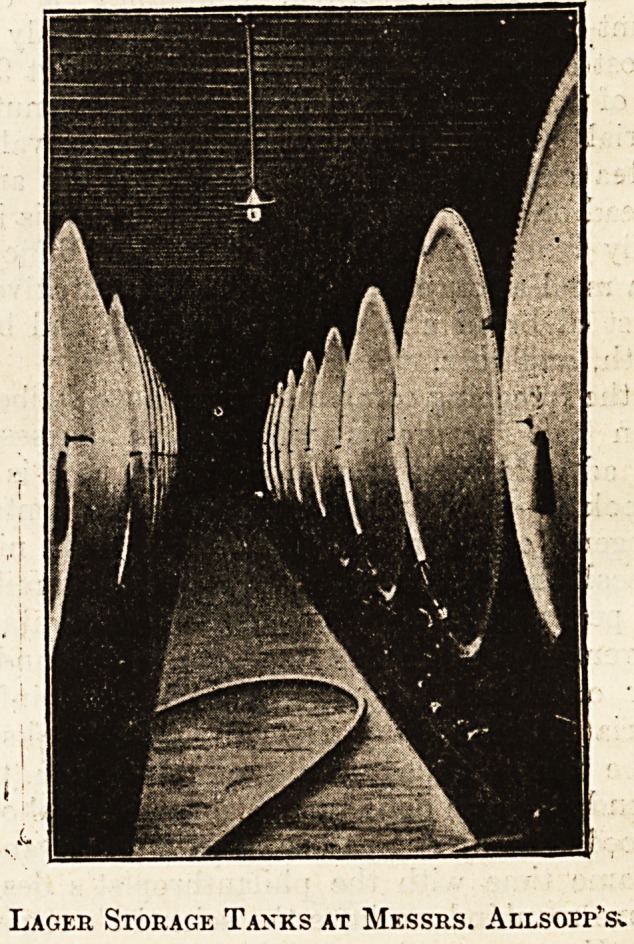# A Special Report on Their Preparation, and Their Chemical, Physiological and Dietetic Properties

**Published:** 1909-04-24

**Authors:** 


### "THE HOSPITAL'S" COMMISSION ON BEERS.

### A SPECIAL REPORT ON THEIR PREPARATION, AND THEIR CHEMICAL, PHYSIOLOGICAL AND DIETETIC PROPERTIES.

### PART I.—THE CHEMISTRY AND MANUFACTURE OF BEER.

We have previously, in two special numbers of The Hospital—April 7, 1906, and June 15, 1907—discussed at length the chemical and physiological properties of Whisky and of Light Wines. Both these alcoholic beverages are extremely ancient preparations of alcohol, but a careful review of the history of the subject tends to show that the alcoholic beverage to which the present report is devoted—namely, Beer—at any rate so far as these islands and Western Europe are concerned, is practically sychronous with the history of the inhabitants.

#### Historical.

The discovery of brewing has been ascribed to Gambrinus, the son of the German King Marcus, who lived 1730 B.C. The historian Pliny mentions the use of beer in Spain and in Gaul, stating that the natives who inhabit the west of Europe have a liquid with which they intoxicate themselves made from corn and water. He further states that this liquid in some parts is so well brewed that it will keep a long time; and finally laments upon the cunning of mankind in being able to gratify their vicious appetites by inventing a method whereby water itself can be made to produce intoxication. Historians in the Middle Ages frequently lament the addiction of the English to drinking, and at this time almost every monastery was famous for the excellence of its ales. One of the earliest spots at which the art of brewing was brought to perfection was Burton-on-Trent. The special fitness of the water at Burton for brewing, which, as a matter of fact, is due to its richness in calcium sulphate, was first discovered by some monks. The sale, however, appears to have been local until about the middle of the seventeenth century, when we begin to hear of Burton ale being drunk at special ale-houses in London. At this time the use of ale as a domestic beverage was practically universal, it being drunk both hot and cold. The introduction of tea, coffee, and cocoa, even when these articles were relative luxuries, at once began to diminish the consumption of ales. The new beverages also exerted an influence upon the quality of the ale drunk, as from this time forward the consumption of the old strong ales began to diminish and that of the lighter ales to increase.

#### Beer Defined.

Beer may be best defined as an alcoholic beverage, produced by the fermentation of a wort derived from malt and other cereals or substitutes, and hops. Beer has two characteristics which distinguish it sharply from either spirit like whisky, or wines. It contains much less alcohol than wines—roughly, less than half as much, and *a fortiori* much less than whisky. Beer also contains a considerable proportion of nutritive substances other than alcohol, which is practically not the case with wines and spirits. This latter difference can be put strongly, and one will be fairly correct in saying that where, as in the case of some wines and all spirits, the only main material is alcohol, in the case of beer only a small part of its nutritive value is represented by its alcoholic content. An exception to this is perhaps provided by certain old specially brewed ales, the alcoholic content of which may run up high in proportion to their total solids. These ales, however, are generally drunk as wines, and are distinctly vinous in character.

#### Wines and Beers Contrasted.

Speaking generally, it would be correct to say that average beer contains half as much alcohol, and twice as much other nutritive material, as wine. The nutrient material contained in beer differs not only in quantity, but also in quality, from that present in wine. In wine the so-called extract or solid matter, or matter other than alcohol and water, consists mainly of nutritive substances containing carbon and hydrogen only, whereas in beer a not inconsiderable proportion of the solid matter consists of substances containing nitrogen, and indeed of albuminous or proteid substances.

A further difference between wines and beer is in relative acidity. This difference is both quantitative and qualitative. The acidity of beer is, roughly speaking, generally about one-fifth to one-tenth that of wine. It is further due to a different cause. The acidity of wine is for the most part due to acids and ethers of the fatty acid series, whereas the acidity of beer is due mostly to acid phosphates. A substance imparting some of its acidity to beer, and not without interest from the standpoint of modern therapeutics, is lactic acid. This substance occurs to a very slight extent in wine; if, however, in wine its quantity becomes considerable the wine is spoiled. In beer it is, in relatively considerable quantities, a normal constituent. The acid moiety in beer consisting of acid phosphates requires some further consideration physiologically. Although the acid phosphates are *ex hypothesi* acid in reaction, they play physiologically a curious *rôle*, in that they have the power of fixing free acid, and being thus converted into phosphoric acid—a drug much used as a tonic medicine, and of considerable value. We have said elsewhere that we think nowadays the fetish of mathematical nutrient value is being unduly emphasised, and when all is said the public will have in the main what it likes, and in a considerable number of cases what it likes is what is best for it. Nevertheless, we can summarise our introductory remarks by saying that beer is *par excellence* the nutritive alcoholic beverage. The alcohol forms only part of its value, and the proportion of alcohol any given beer contains in no sense measures its strength. It is possible with some well-made lager beers to have a full nutritive beer combined with a low alcoholic strength. When a man drinks good beer he drinks and eats at the same time, just as when he eats a bowl of soup. The terms eat and drink are curiously but inconsistently used as connoting the difference between what is merely quenching our thirst and what is actually consuming nourishment. A man might more appropriately be said to eat beer than to eat certain kinds of soup, or indeed water-melon.

There are many varieties of beer on the market. Speaking roughly, the value of a beer is proportional to its original gravity, and increase in alcoholic strength should always go with increase in original gravity, though the converse is not true, as there are some good lager beers on the market with a high specific gravity and a low alcoholic strength. The main varieties of beer are the so-called mild ales, the bitter ales, the black beers (stout and porter), and lager beer. The general technique of brewing is the same for all these varieties, and we shall now proceed shortly to describe it, not with a view to enable our readers to brew for themselves, but to tell them how beer comes to be what it is.

### Malting.

The first essential process in brewing is malting. Malt is germinating barley, or, in common language, sprouting barley. Barley during germination develops several ferments. The biological *raison d'être* of these ferments is so to alter the constituents of the grain as to make them assimilable by the growing plant. The most important of these ferments is diastase, or a ferment which renders the starch of the grain soluble. In addition, ferments which convert insoluble into soluble proteids are also present, the so-called proteolytic ferments. The growth of the germinating grain is regulated by the extent to which the whole grain is moistened or steeped in water, and also by the temperature to which it is subsequently subjected, or, in other words, by the temperature of the malting floors. It may be taken generally that the size and nature of the sprout from the grain is a measure of the degree to which the glycolytic and proteolytic processes have progressed in the substance of the grain itself.

Now the brewer does not want to grow barley, but he wants to utilise the effect of the sprouting of the barley grain in making the substance of the grain suitable material from which to make beer. If the germination were allowed to proceed indefinitely all the starch of the grain would be converted into maltose, and this would be used up by the growing plant, for which, of course, it was originally intended. The brewer does not want this to happen, as he wishes his beer eventually to contain dextrine, a substance intermediate between starch and maltose. When, therefore, he thinks he has got what he wants, he stops the process of germination. He does this by dry heat. In considering the question of brewing it must never be forgotten that the brewer, unlike the distiller, does not want to make practically pure alcohol. Technically the destruction of the germinating grain by heat is termed kilning. The flavour eventually possessed by the beer, as well as the amount of nutritive material other than alcohol which it will contain, is closely related to the exact manner in which this process of kilning is carried out. The malt is first dried and then heated to various temperatures. The dry heat kills the germinating plant, but only modifies the unorganised ferments present in the grain. These ferments, like the pepsin of the gastric juice, are capable of withstanding certain temperatures with only a temporary suspension of their activity, and can when subsequently placed under suitable conditions (moisture and temperature) re-exert their ferment action. The first stage in kilning is drying the malt, and this should be done at as low a temperature as possible. It is carried out on the kilning floor, which usually consists of perforated metal or tiles, the hot air being made to pass through the perforations. The next stage is to raise the temperature to a degree sufficient actually to kill the germinating grain. Not only is the germinating grain killed by kilning, but also the activity of the ferments, especially that of the diastatic ferment, is modified.

The brewer does not want a too active diastase, because, as will be seen later on, when he submits the malt, after grinding it, to mashing he does not want all its starch converted into sugar, but wishes to retain in his beer some of the bodies intermediate between starch and sugar, or, in other words, nutritive material not capable of being directly converted into alcohol by the subsequent treatment with yeast. The distiller has quite another object in view. In his mash he wants all the starch converted into sugar in order that by subsequently pitching with yeast he may obtain a maximum yield of alcohol. The brewer achieves his object, and obtains a not too active diastase by kilning at a higher temperature than the distiller and thereby reducing the activity of his diastase.

We are describing this small point of technique with a view of emphasising to our readers the fact that the actual presence of alcohol in beer is no more important than the presence of other nutritive elements, including starch derivatives, sugar, and intermediate products. Alcohol might quite well be regarded as a by-product in brewing, whereas it is practically the only product in distilling.

After drying and kilning the malt is brought to a fine state of mechanical division by crushing, and is then ready for the second process in brewing—namely, mashing. In brewing black beer or stout a portion of the malt to be used for the mash before being crushed is roasted until it is of a dark brown colour. In ordinary stout making about 5 per cent. of this crushed roasted malt is used for the mash, and it is to this moiety of the mash that the dark colour and burnt sweetish taste of stout and dark beers are due.

### Mashing.

For this purpose the crushed malt is conveyed by mechanical means to a large vessel termed the mash-tun. Thanks to Messrs. Bass, Ratcliffe and Gretton, we give a photograph of what is known at their brewery as the King's mash-tun, being actually the mash-tun in which the mash was made when His Majesty visited their brewery. The mash-tun is provided with a perforated bottom. When the crushed malt has reached the mash-tun successive doses of hot water are added to it. The average temperature of this water in English breweries is from 140° to 160° F., although the final dose of water is often at a higher temperature than this. The nature of the water used for mashing—*i.e.* brewing—is by no means a matter of indifference, and the original pre-eminence of Burton as a brewing centre was probably mainly due to its water being, on account of its mineral constituents, especially suitable.

Burton water is hard, and contains a high proportion—77.87 grains to the gallon—of calcium sulphate. The exact reason why a water of this nature is so suitable for brewing is not known, but it is interesting in this connection to note the manner in which recent research has emphasised the importance of calcium salts in catalytic processes caused by organised or unorganised ferments. In the process of brewing ferment action plays an all-important *rôle*, and it is probably in influencing favourably the activity of the ferments that the value of water rich in calcium sulphate consists.

It will be remembered that we left the malt at the completion of malting, having been dried and heated to a considerable temperature; in other words, with the germ killed and the activity of the ferments diminished and their actions suspended for want of moisture. By placing the malt in the mash-tun we once more place it in condition under which its ferments can again get to work. Two important changes at once begin to occur in this mixture of crushed malt and water. The starch of the grain becomes mainly converted by the resuscitated diastase into dextrine and maltose and a series of intermediate bodies. At the same time the insoluble proteids contained in the crushed malt become acted upon by the proteolytic ferments and become converted into soluble proteids, mostly albumoses and peptones.

Before proceeding to follow the resulting sweet wort, as the finished product of the mash-tun is called, we must pause to consider an interesting by-issue upon which much depends. As a matter of fact, ground malt, when treated with water as we have just described, contains more diastase than is required to convert its individual starch into dextrine and maltose, yet nevertheless in the best beers all the sugar contained in the sweet wort is derived from the malt of the mash, and consequently all the alcohol contained in the finished beer is thus derived. Beer production can be rendered more economical, however, if the sugar content of the sweet wort be increased either by adding sugar itself to the mash-tun, or by adding starch which will be converted into sugar by the excess of diastase present in the ground malt. By this means the amount of alcohol derived from a given weight of malt can be very largely increased. Beer thus produced is said to be derived from substitutes. The resulting beverage, if made from sound materials, is perfectly wholesome, but is essentially an inferior article to beer manufactured entirely from malt.

To return to the process of brewing where we left it, we have in the mash-tun a solution containing for the most part maltose, dextrine, and soluble proteids mixed with crushed malt. The next process is essentially simple filtration; the clear liquid is filtered through the malt *débris*, and this solution, varying as it does in colour according to the proportion of roasted malt used in the mash, is now submitted to the next stage of brewing, namely, hopping.

### Hopping.

From the mash-tun the sweet wort is pumped into the "copper," a large copper vessel. What essentially happens in the copper is that a decoction of hops is made with the sweet wort, or solution of dextrine, maltose, and certain proteids, as the extracting agent. A strong or a weak decoction may be made, much or little hops being added according to the desired characteristics of the finished ale. Some of the heaviest hopped beers are the black beers or stout, hops being added to these beers in as high a proportion as 6 lbs. to the barrel.

The King's Mash-tun.

Hops as used for brewing consist of the specially dried and preserved unfertilised flowers and flowering tops of the hop plant, *Humulus lupulus*. The most important morphological constituent of the hops is the so-called lupuline. This is a bright yellow powder, and is actually a dried secretion from the glands of the hop. In this lupulinum are contained the active principles of the hops in greatest quantity, and as is probably well known to our readers, lupulinum is actually official in the British Pharmacopœia, and is mostly given in two to five grain doses in the form of a pill. The main active principle of the hop is a volatile oil. This volatile oil is chemically a mixture of hydrocarbons and valerol; by keeping the hop this latter undergoes oxidation to valerianic acid, and this gives to hops which have been kept a peculiar and characteristic bouquet. The next active principle of importance is the hop resin, and with this is associated a bitter acid principle; a further substance of interest, according to some authors, is a basic body of alkaloidal nature, so-called hopein. Hopein appears to have a narcotic action allied to morphine.* A special form of tannin, so-called hop tannin, is also contained in hops, and this as will be seen later is of considerable importance in the technique of brewing.

It would be going beyond our purpose here to enter fully into the methods of drying and preserving hops, but they require extreme care if their bouquet is to be retained; as a rule in breweries they are kept in cold storage. We have entered

A Corner of one of the Union Rooms at Messrs. Bass's Brewery.

so far in detail into the properties of hops because it must be emphasised that while beer derives its dietetic properties from its dextrine, sugar, and alcohol content, *i.e.* from the malt, its medicinal properties are derived from the hops.

To return then to the hopping copper or the vessel in which the sweet wort is boiled with hops, three important changes happen to the future beer in this vessel. First it is boiled: this checks all further conversion of starch or dextrine into sugar; it also renders the wort sterile, killing all stray bacteria; and finally, it precipitates part of the albuminoid constituents. The second change that happens to the wort in the hopping copper is that the boiling extracts the active principles of the hops and brings them into solution. The third change is that certain constituents of the wort become acted upon by the hop tannin. These substances are mainly the albuminoids, and these become for the most part precipitated. After treatment in the hopping coppers the wort becomes darker in colour, but clearer, still remains acid, and becomes possessed of a strongly bitter taste, and is known as the bitter wort, and should be quite sterile. Before being submitted to further treatment the bitter wort is filtered through the hops. The next process which it undergoes is cooling, and this is done in various ways. In some breweries it is cooled at once over a refrigerator and in others slowly in cooling tanks. The advantages of cooling in large open vessels are that the wort becomes aerated and that certain further precipitation of insoluble products takes place. The disadvantage, however, is that the wort becomes exposed to reinfection of stray organisms, although in modern breweries this rarely happens, and even after exposure to the air in the cooling tank the wort remains sterile, chiefly, no doubt, owing to its acidity and the antiseptic properties which it has derived from the hops.

* Recent research has shown that hops also contain morphine.

### Fermentation.

The wort as it leaves the hopping coppers, or rather the cooling tank, contains the same amount of fermentable sugar as when it left the mash-tun. The next object of the brewer is to ferment nearly all this sugar into alcohol. For this purpose the wort, which has a certain specific gravity, is conveyed into the fermenting backs or squares and is there pitched with liquid yeast or barm. The sugar in the wort is broken up by the yeast, and alcohol and $CO_2$ are formed in the process, the specific gravity of the wort being correspondingly reduced. The technique of the fermentation process in brewing is complicated, fermentation actually taking place in two stages, the wort during the second being at the same time separated from the frothy yeast, which has, of course, in the first stage multiplied enormously. In the making of English beers, as distinguished from lager, the total period of fermentation is about five days. It is very important during fermentation to regulate the temperature, and in English beer the wort usually goes into the fermenting backs at about 60° F., and during fermentation its temperature rises to about 75° F. In the case of stout the specific gravity of the wort is about 1,075 when fermentation begins and about 1,022 when it is complete.

The fermentation of lager beer is essentially different, and we shall describe this separately. Considerable difficulty is experienced in separating the fermented wort, or beer as it really is, from the frothy yeast, and to effect this purpose many appliances have been designed. The one most generally used at Burton is that known as the Burton union system. We give a photograph showing this process at work. After the first stage of fermentation is complete, which is ascertained by the gravity of the wort, the fermented wort is allowed to flow into a series of barrels, so mounted that they rotate axially; these barrels are fitted with a cooling apparatus, and also with a pipe and cock at their base for the removal of the finished beer. The striking feature about them is that at their upper surface they are provided with long tubes shaped like a swan's neck and fitting into the barrels through a bung-hole; these necks all bend over and give into a common gutter. The frothy barm, by virtue of the pressure behind and its lightness, eddies up through the swan necks and drops over into the common gutter, where it settles at the top. It must not be forgotten that fermentation is proceeding the while, and continues until this cleansing process is completed and, indeed as will be mentioned later, afterwards.

When the barm has separated, the beer in some breweries is allowed to settle in a settling tank, but in the majority of cases it is at once racked off into storage vessels or large vats, or trade casks as they are called. Very frequently a further dosing of hops is given to the beer in these casks, and this process is known as dry hopping. The best qualities of ale are allowed to remain in cask for varying periods from six weeks to three months. While in cask their alcohol strength slightly increases owing to the conversion of more sugar into alcohol, and they become bright and sharp owing to the absorption of the accompanying $CO_2$. The cheaper varieties of beer do not remain in store, but are sent out of the brewery within ten or fourteen days of mashing. These latter are last of all subjected to what is known as priming and fining.

### Priming and Fining.

Priming consists essentially in adding a small quantity of sugar solution to the beer in cask. This sugar becomes rapidly fermented into alcohol and $CO_2$ by the active yeast which remains, and this last fermentation gives the beer an extra bright appearance and a sharp taste. Finally, some beers are fined before being sent out. The fining reagent is usually isinglass solution; this combines with the tannic acid compounds of the beer and causes a precipitation, and thus any insoluble or opaque-looking substances which may be floating in the beer are carried down. The barrels are subsequently rolled and the precipitate allowed to ooze out by the action of gravity. Beer as originally brewed was invariably of a muddy or opalescent appearance, but this, so long as beer was drunk in metal tankards, was not noticed by the consumer.

Nowadays, however, as beer is almost always drunk in glass, the public demand that the beer should look bright and transparent. Since beer in this country has to be kept at temperatures very apt to foster secondary undesirable fermentation, a preservative is almost universally added to the beer before it leaves the brewery. This preservative is generally a solution of so-called bisulphite of lime. This is practically a solution of $SO_2$. Modern research has shown that the $SO_2$ so added combines with the aldehydic substances present in the beer, and forms with them compounds which, in the quantity in which they are present, are quite harmless.

### Bottled Beers.

In order to meet the exigencies of the retail trade at home and the enormous export trade, a very large quantity of beer is bottled. Beer to bottle well should be specially brewed, and should be more heavily hopped and stronger than ordinary draught beer, and further, it should be longer stored, or in other words should have undergone a more profound secondary fermentation. By this its alcoholic strength is somewhat increased; its content of the antiseptic principles of the hop is also somewhat increased; these two properties render it in a better condition to keep. Further, the $CO_2$ which it gradually absorbs during the secondary fermentation gives it a bright and sparkling appearance and a sharp and pleasant taste. All good bottled beers owe their properties to these qualities, and some of the bottled beers of the best makers, even after ten years in bottle, have a peculiarly pleasant and refreshing taste.

Unfortunately, however, the demand for cheap bottled beer has placed upon the market a very inferior article, from both the gastronomic and dietetic standpoints. This variety of bottled beer is produced by what is known as the chilling and carbonating process. For this product ordinary draught beer is taken, and when it is ready for racking is rapidly "chilled" or reduced to a very low temperature. This causes rapid deposition of suspended matter, which would under ordinary circumstances subside gradually during maturation. The beer is then bottled at a low temperature, with the result that it at once assumes a bright clear appearance. Subsequently, artificially produced $CO_2$ gas is pumped into the beer under pressure. Beer bottled according to this method when poured out looks bright, clear, and sparkling but is invariably thin, and when consumed is very apt to cause indigestion, owing to the fact that when it is submitted to the temperature of the stomach a considerable evolution of gas takes place; this not only produces distension and flatulence, but also actually irritates the gastric mucous membrane.

Those who have taken a $CO_2$ bath at Nauheim or Spa know how the bubbles of carbon dioxide gas liberated from the solution of the bath on the surface of the skin redden and irritate it. The mucous membrane of the gastro-intestinal tract is far more delicate and susceptible to irritation than the skin, and we know as a fact how solutions saturated with carbon dioxide under pressure will produce redness, congestion, and finally irritation of it. Just as thin or watery highly saturated solutions of carbon dioxide gas are irritants to the gastric mucous membrane and inhibit the secretion of the digestive juices and cause the transudation of mucus, which is actually harmful to the digestion, just so do viscid solutions containing a small quantity of carbon dioxide, which under the influence of the heat of the stomach is gradually evolved in a gaseous state, act as gentle and efficacious stimulants to the secretion of the digestive juices and to the movement of the stomach and thus act as aids to the digestion. In the case of viscid solutions the viscidity acts as a cushion between the bubbles of carbon dioxide gas and the gastric mucous membrane, and thus converts what would be a pathological irritant into a physiological stimulant. This is precisely what happens in the case of good bottled beers which only contain a moderate quantity of carbon dioxide gas, and which are full bodied or viscid owing to their richness in extract (dextrine and sugar) as compared with chilled and carbonated bottled beer. These latter are thin like water and contain an inordinate quantity of carbon dioxide gas. We have entered into this subject in some detail because we consider it of importance, and feel convinced that the bad reputation beer has earned in some quarters as a dietetic beverage on account of it causing, when taken with meals, flatulence and gastric discomfort, is due to the fact that the beer in question was one of the thin chilled and carbonated bottled beers described above.

### Lager Beer.

The process we have described above is that of ordinary English brewing; German or lager beer is, however, brewed on a different principle. The characteristic of good lager beer is that it contains a

Refrigerating Plant at Messrs. Allsopp's Lager Beer Brewery.

high extract, and has a creamy mucilaginous effect on the palate, as well as a high nutritive value, and at the same time has a very low alcoholic strength. Dextrine and sugar solutions, even if they are heavily impregnated with the antiseptic principles contained in hops, are nevertheless relatively unstable, and especially is this the case if, as in lager beer, there is not present an adequate quantity of alcohol to assist in preserving the product. From this it follows that, however careful the brewer of ordinary English ale ought to be to prevent contamination with stray bacteria, the brewer of lager beer must be even more careful.

Hence, as we shall see in describing briefly the technique of lager beer brewing, the greatest possible care is taken to render and keep the beer sterile throughout the whole process, and to still further pasteurise it in bottles. The main difficulty of the lager beer brewer in the past has been to produce a light and first-class beer sufficiently stable to fulfil without the aid of objectionable methods of preservation, the exigencies of the retail trade and reach the consumer in sound condition; Messrs. Allsopp have succeeded in doing this; we are indebted to them for the practical information which has enabled us to give briefly to our readers an account of the brewing of lager beer in this country. The great German lager beer breweries export large quantities of lager beer to this country, but in order to make quite sure of the product in question reaching the consumer in apparently good condition they often dose their export beer with a preservative—as a rule salicylic acid—and by no means always in unobjectionable quantity.

The technique of lager beer brewing differs in many important respects from that of ordinary English brewing. In the first place English beer is produced by what is known as the infusion and top fermentation method. These attributes are derived from the facts that in English brewing the mash is made by adding hot water to the grist, and the yeast during the fermentation process rises to the top. In lager beer brewing the crushed malt is mixed with cold water, and then a certain quantity of hot water is added until the temperature of the whole is about 60° F. Successive portions, about one-third at a time, of the mash are then transferred seriatim to another vessel called the mash copper, and here they are boiled. After each third has been boiled for a varying length of time it is returned to the mash tun. Matters are so arranged that the temperature in the mash tun never exceeds, but finally reaches about, 150° F. The resulting sweet wort is then filtered as in the English method, and at once transferred to the hopping coppers. It usually enters these coppers at about a temperature of 160° F.

It is advisable to pause here for a moment and consider in what way the manipulations practised in lager beer brewing affect the chemical composition of the wort. The successive boilings tend to produce three main results. They hamper the action of the diastase, so that in lager beer wort more dextrine and intermediate bodies are present in proportion to the maltose than in English wort. Further, those albuminoids coagulable by heat are more completely removed, the resulting wort being thereby clearer. The third change is that more of the original proteids are hydrolysed, or, in other words, converted into peptones and albumoses. As we shall subsequently see, the final product is relatively richer in these substances than English beer. But in the case of lager beer there is a very special reason for hydrolysing the proteids of the mash more completely than in the case of English beer. This reason is that the resulting wort makes a better nutrient medium for lager yeast.

The next process is hopping, and this, as in English breweries, is done in the hopping coppers. When the bottom of these are well covered with wort, hops are added. The contents of the copper are then boiled for an hour. More hops are then added, and the boiling process repeated. As the bouquet-producing substances in the hops are volatile, the best hops are, as a rule, added last, in order to avoid loss of bouquet by evaporation. The average time for boiling ordinary lager beer is about two hours, but export beer is boiled about half an hour longer. The quantity of hops added varies considerably, but, speaking generally, English beer is more heavily hopped than lager.

The bitter wort thus produced is now cooled as rapidly as possible, not in cooling tanks, but over a refrigerator. This method of rapid cooling is adopted in order to run the least possible chance of the wort not remaining sterile. As a matter of fact, only one hour and a half is allowed to elapse between leaving the hopping copper and pitching with yeast.

The process of fermentation adopted in the manufacture of lager beer is that known as bottom fermentation, because the yeast during fermentation does not rise to the top, as in the case of the English method, but falls to the bottom. Biologically, lager beer fermentation is characterised by the fact that it takes place at a relatively low temperature, and is continued for a relatively long time. It takes place in two stages—namely, in the fermenting backs and in the store barrels. The initial temperature at which fermentation is begun in the fermenting backs is 47° F. The beer remains in the backs from 9 to 11 days, and the maximum temperature reached is 52° F. This occurs about the middle of the fermentation. In order to prevent infection by stray bacteria all air is filtered. Formerly fermentation took place in vacuo, but this method has now been generally abandoned. The beer is separated from the yeast in the fermenting backs, and is then pumped into the store barrels, where it is allowed to remain at a very low temperature for three months. In the store barrels further fermentation takes place. After it has been in store for three months it is carefully filtered; part of the filtering plant consists of glass vessels, during its passage through which the beer can be observed. This is necessary, as it is interesting to note that the beer is actually never seen from the time it leaves the refrigerator until it appears, some three or four months afterwards, in the glass parts of the filtering plant. After filtration the beer is bottled or casked as may be required. All beer bottled at the brewery is pasteurised in bottle. In the case of the beer bottled elsewhere the barrels are conveyed in refrigerating vans to the bottlers.

### Stout and Porter.

The last kind of beer which we have to consider is stout or porter—porter simply being a weak stout.

The largest makers of stout, and indeed the largest brewers in the world, are Messrs. Guinness, who make, annually, stout paying a duty of approximately one million sterling. We shall consider the dietetic properties of stout elsewhere, and so far as concerns the technique of its production we have little to say, since it differs only very slightly from that of beer. The dark colour of stout is due to the fact that roughly 5 per cent. of the malt used for the mash is previously roasted. The hopped wort is cooled by means of a refrigerator, and is at once submitted to the alcoholic fermentation, which, as in the case of beer, partly takes place in the fermenting back and partly in the skimmer or the apparatus in which the yeast is separated from the beer. After the stout leaves the skimmer it remains for twelve hours in what is called the settling vat, and is then transferred to the storing vat. The weaker varieties of stout, or so-called porter, are practically only consumed locally. These do not undergo storing, and are drunk within 14 or 21 days of mashing. So-called extra stout remains in the storing vat for about 14 days, and should be consumed within two months of bottling. Export stout remains in the storing vat for a year, and will keep good for years in bottle. This variety of stout is much richer in alcohol than the others, and is also more heavily hopped, about

Lager Storage Tanks at Messrs. Allsopp's.

6 lb. of hops being added per barrel of stout. London stout is produced in practically an identical manner, and, as will be seen from our analyses, is practically an identical article. An interesting difference in the technique of stout and beer brewing is that, as we have seen above, beer requires an initially hard water, whereas stout requires an initially soft water.

### Beer Compared with Tea and Beef-tea.

In the preceding section we have endeavoured to show how beer comes to be what it is, and it now remains for us to consider more exactly what it is. We cannot do better for the moment than compare beer to tea or coffee. Of every cup of tea that we take about 99 per cent. consists of water, the remaining part of drugs—namely, an alkaloid caffeine and an astringent principle tannin, and an aromatic principle or volatile oil. Of every glass of beer that we take from 89 to 94 per cent.

consists of water, the remaining 11 to 6 per cent. consisting of from 2 to 6 per cent. of alcohol, of from 1 to 5 per cent. of maltose, or malt sugar, from 2 to 3 per cent. of dextrin (the nutrient material contained in toast and biscuits and bread crust), and 0.5 per cent. of proteid or nutriment of the nature of meat. A cup of tea, including the milk and sugar, with which it is mostly drunk by the lower classes, costs about ¾ of a farthing, and is sold for anything up to 3d.; a glass of beer costs about two farthings and is sold from 1d. to 2d. or 3d.

As the view that beer has no nutritive value in itself and merely consists of a beverage upon which a certain portion of the community intoxicates itself is somewhat prevalent, we will finally compare beer to another beverage which has great repute as both a food and a stimulant—namely, good home-made beef-tea, containing a certain amount of meat residue, produced at roughly about the cost of 9d. a pint. This contains about 96 per cent. of water and about 2 per cent. of real nutritive material. It is true that the nutritive value of beef-tea can be greatly augmented by the amount of bread or toast consumed with it, but this is also equally true of beer, and it would be difficult to find a meal at once simpler and more nutritive than a crust of bread and cheese, or bread and butter, or both, and beer.

In the foregoing comparisons we have deliberately chosen two beverages which each possess the same advantage as beer in a respect that is often overlooked—namely, their chance of containing pathogenic organisms. This is practically nil. If we recall the epidemics caused by polluted milk, we shall perhaps grasp more clearly the advantages of a beverage prepared as tea and coffee and beer under conditions rendering pollution by infective bacteria extremely improbable. Our remarks must not be misconstrued; we are quite alive to the tremendous harm done by the abuse of alcohol, and the good done by sympathising with temperance; at the same time with the philanthropist's desire to improve mankind we have the scientist's regard for fact. Upon untruth no enduring fabric, however philanthropic its motive, can ever be securely built.

The fact is that good, properly made beer is a beverage containing a very small amount of alcohol and a relatively large amount of nutritive material. All beverages because they contain alcohol should not be regarded in the same light. The spirit nipper is committing quite a different act from the beer drinker; in fact, beer is much farther removed from the point of view of its alcohol content from some wines and all spirits than ginger-beer is from beer. The Revenue authorities exempt from duty any beverage containing under 2 per cent. of proof spirit, or, roughly, 1 per cent. of alcohol. Ginger-beer usually contains this amount, and koumiss, a beer made from milk by fermenting its sugar of milk with a special kind of yeast, contains slightly more. There are beers on the market containing 2 to 3 per cent. of alcohol.

### Consumption of Beer.

Beer is very largely and very generally consumed all over the civilised world. The Japanese consume a variety of beer made from rice. The production and consumption of beer in the United States has increased enormously during the last decade. The country which consumes the greatest amount of beer per head of population is Bavaria, the consumption there being reckoned at 40 gallons per head per annum. In the United Kingdom the *per capita* consumption has during recent years shown a distinct diminution. The average consumption per annum per head, for instance for 1887-1897 was 31 gallons, for last year it was 27 gallons, or little more than half that consumed in Bavaria. In the city of Munich, which is, so to speak, the German Burton, the consumption per head is very large, being 70 gallons per head per year, or about 1½ pints per day for every man, woman, and child.

### Chemical Composition of Various Beers.

For the purposes of this research we have collected samples of beer from various sources and analysed them. Practically all the well-known brands of beer have been included in these analyses. We give below a table showing the results of these analyses.

As in the case of wines, there is no absolute chemical standard for what is a good beer or what is a bad beer, and one infers that a certain beer is good or

TABLE OF ANALYSES.

| No. | — | Original Gravity | Alcohol by Weight | Total Solids | Ash. (Mineral Matter) | Maltose | Dextrine | Nitrogen | Phosphoric Acid | Total Acid | Volatile Acid | Fixed Acid | Maltose. Dextrine Ratio | Solids. Alcohols Ratio | Attenuation | Residue. Total Solids −(M+D) | No. |
|---|---|---|---|---|---|---|---|---|---|---|---|---|---|---|---|---|---|
| | | | % | % | % | % | % | % | % | % | % | % | | | % | % | |
| 1. | Allsopp's Lager (bottled) | 1,044·8 | 3·88 | 4·14 | 0·33 | 1·02 | 1·56 | 0·068 | 0·021 | 0·086 | 0·059 | 0·027 | 1 : 1·5 | 1 : 0·93 | 64·3 | 1·56 | 1 |
| 2. | { Ditto (draught) | 1,052·1 | 3·45 | 6·85 } | 0·23 | 2·03 | 3·27 | 0·088 | 0·045 | 0·092 | 0·048 | 0·044 | 1 : 1·59 | 1 : 0·50 | 47·3 | 1·59 | 2 |
| | { Ditto Dark | 1,046·1 | 3·69 | 4·81 } | | | | | | | | | | | | | |
| 3. | Ditto Light | 1,046·9 | 4·01 | 4·38 | 0·13 | 1·1 | 1·60 | 0·077 | 0·029 | 0·082 | 0·033 | 0·049 | 1 : 1·45 | 1 : 0·91 | 63·9 | 1·68 | 3 |
| 4. | Mild Ale (draught) ... | 1,043·4 | 3·88 | 3·78 | 0·30 | 1·44 | 0·95 | 0·044 | 0·038 | 0·090 | 0·037 | 0·053 | 1 : 0·66 | 1 : 1·03 | 66·3 | 1·40 | 4 |
| 5. | Ditto ditto ... | 1,054·1 | 4·24 | 5·80 | 0·49 | 2·83 | 0·70 | 0·067 | 0·037 | 0·104 | 0·044 | 0·060 | 1 : 0·25 | 1 : 0·73 | 58·6 | 2·27 | 5 |
| 6. | Ditto ditto ... | 1,040·9 | 3·60 | 3·76 | 0·33 | 2·01 | 1·28 | 0·068 | 0·079 | 0·118 | 0·064 | 0·054 | 1 : 0·64 | 1 : 0·96 | 64·5 | 0·47 | 6 |
| 7. | Ditto ditto ... | 1,056·8 | 4·46 | 5·96 | 0·30 | 3·33 | 0·60 | 0·077 | 0·061 | 0·088 | 0·018 | 0·070 | 1 : 0·18 | 1 : 0·74 | 59·4 | 2·03 | 7 |
| 8. | Bass's Ale (draught) ... | 1,060·8 | 5·02 | 5·80 | 0·36 | 2·13 | 1·60 | 0·076 | 0·049 | 0·098 | 0·044 | 0·054 | 1 : 0·75 | 1 : 0·86 | 63·1 | 2·07 | 8 |
| 9. | Allsopp's Draught Ale | 1,055·1 | 4·55 | 5·27 | 0·29 | 1·60 | 1·70 | 0·078 | 0·052 | 0·112 | 0·063 | 0·049 | 1 : 1·06 | 1 : 0·86 | 63·0 | 1·97 | 9 |
| 10. | Bass's Pale Ale (bottled) | 1,061·1 | 5·32 | 5·01 | 0·35 | 1·43 | 1·57 | 0·073 | 0·054 | 0·094 | 0·060 | 0·034 | 1 : 1·1 | 1 : 1·06 | 68·3 | 2·01 | 10 |
| 11. | Light Bitter (bottled)... | 1,044·5 | 3·66 | 4·53 | 0·22 | 1·78 | 1·33 | 0·044 | 0·040 | 0·082 | 0·035 | 0·047 | 1 : 0·75 | 1 : 0·80 | 60·4 | 1·42 | 11 |
| 12. | Ditto ditto ... | 1,047·5 | 3·77 | 5·09 | 0·20 | 2·47 | 1·44 | 0·064 | 0·047 | 0·076 | 0·022 | 0·054 | 1 : 0·58 | 1 : 0·74 | 58·1 | 1·18 | 12 |
| 13. | Guinness's Extra { | 1,067·7 | 5·15 | 7·32 } | 0·44 | 2·84 | 2·13 | 0·147 | 0·116 | 0·140 | 0·065 | 0·075 | 1 : 0·75 | 1 : 0·70 { | 58·2 | } 2·35 | 13 |
| | Stout (bottled) { | 1,068·5 | 5·20 | 7·87 } | | | | | | | | | | | 58·7 | | |
| 14. | Stout (bottled) ... ... | 1,074·7 | 5·04 | 9·26 | 0·36 | 5·66 | 0·58 | 0·130 | 0·078 | 0·119 | 0·025 | 0·094 | 1 : 0·10 | 1 : 0·54 | 52·0 | 3·02 | 14 |
| 15. | Guinness's Extra Stout (draught) } | 1,074·7 | 5·70 | 7·89 | 0·33 | 2·18 | 2·82 | 0·141 | 0·110 | 0·157 | 0·059 | 0·098 | 1 : 1·3 | 1 : 0·72 | 59·2 | 2·89 | 15 |
| 16. | Stout (draught) ... | 1,091·4 | 6·23 | 11·20 | 0·45 | 5·66 | 2·32 | 0·074 | 0·102 | 0·162 | 0·065 | 0·097 | 1 : 0·41 | 1 : 0·56 | 52·7 | 3·22 | 16 |

bad; as in wine, not from the quantity of any or all the constituents present, but from their proportion. Beer is a light alcoholic beverage, hence it should not contain much alcohol. The light beers should contain from 3 to 4 per cent., the bottled ales from 4 to 5 per cent., and the stouts from 5 to 6 per cent. Alcoholic strength should also increase *pari passu* with body, that is with total solids and flavour. Beer should froth well, and should leave a mucilaginous creamy sensation on the palate, and should be dry, not sweet, and not sharp in taste. These latter qualities depend upon the presence in beer of a proper proportion of dextrin, and of the ratio between this substance and the maltose present. In the best ales these two substances are present in approximately equal amount. A reference back to the description of the different processes of brewing will show how the proportion of these two substances can be varied. Dietetically a beer containing any considerable excess of maltose over dextrin is apt gastronomically to be mawkish, to lose its creamy mucilaginous effect on the palate, to be, volume for volume, less thirst quenching and more bilious. From these considerations arises the importance of the maltose-dextrin ratio as a criterion of good beer. The head or froth which forms on beer when it is poured out should not be of the nature of effervescence, but of actual froth, and should have a certain consistency and remain for a definite time. Those beers which have been bottled by the chilling and carbonating process give always a gaseous and bubbling head when poured out, and these beers we do not regard as sound from the gastronomic standpoint. Beer should not taste either sour or flat, but should have a good round flavour according to its kind (bitter or mild) of hops. Stouts have a special sweet and burnt taste, which many people like, and which is produced by the use of roasted malt. They should have more body than beer; their fulness of body and their "head" is a criterion to the experienced that their flavour is actually produced by roasted malt, and not artificially. Otherwise they must be judged by the same standard as beer. As we have mentioned before, the chief varieties of beer are the mild ales, the bitter ales, the black beers or stout and porter, and lager beer. Each of these must be judged on its own merits, and we shall, before criticising the actual beers we have examined, just consider their main characteristics.

*The mild ales* are the beers consumed mostly by the working classes, and exhibit a great variety as regards quality and gravity. Thus the ordinary London mild ales vary between a gravity of roughly 1,040 to 1,055. In Burton and in the Midlands, on the other hand, mild ales are produced with a much higher gravity. The ordinary mild ale of the working man in those districts runs up to a gravity as high as 1,072. Generally speaking, the mild ales contain proportionately more maltose to dextrin than bitter ales, and are possessed of a full sweet taste; the alcohol, as a rule, is not high. *The bitter ales* may be subdivided into two classes—namely, first, the light bitters and family ales of a gravity varying between 1,040 and 1,050, and the pale and stock bitter ales, which have a gravity of from 1,055 up to 1,075. Bitter ales are made both for draught and for bottle. In their manufacture a finer class of material is employed, and they are of a more dextrinous and drier character than the mild ales. As a rule the proportion of alcohol and solids in them is higher than in the mild ales; they also contain more hops.

With regard to the *black beers*, these may be subdivided into stouts and porters or coopers. Broadly speaking, stout is a superior porter, or *vice versa*. As a rule the gravity of the stouts is very high, varying from 1,065 to 1,090, and even more. The gravity of porter is, as a rule, in the neighbourhood of that, or rather above that, of mild ales. Stouts are made both for draught and bottle, and here again we have distinct varieties, such as the Irish stouts and the London stouts.

*Lager Beer.*—There are two main kinds of this class of beer manufactured in the United Kingdom—namely, the Munich or dark type, and the Pilsner, or light-coloured, variety.

The characteristics of lager beer are a relatively low proportion of alcohol and a high percentage of solids. Lager beer is also relatively rich in proteid constituents owing to the occurrence during brewing of a high degree of proteolysis (peptonisation), all proteids which are not hydrolysed being precipitated by the heating processes, and subsequently removed by filtration.

### Criticism of the Beers Analysed.

1. *Light Beers.*—We will deal first of all with the light popular beers of the cheap variety, such as would be consumed by the masses. In the case of No. 4 the gravity is very low; the alcohol is higher than the solids, and there is a high maltose content. The nitrogen is also decidedly low. As might have been expected the attenuation is high. It is probable that in the brewing of this beer a considerable quantity of malt substitutes were used. In the case of No. 6 the gravity is also very low, the maltose preponderates largely, and there is a very small residuum of extract when the dextrin and maltose are subtracted from the whole. Here, also, probably a large quantity of malt substitutes have been employed.

Nos. 5 and 7 are fair examples of good-class London mild ales. In both there is a considerable excess of maltose over dextrin.

Taking next the Light Bottled Beers, it will be seen that No. 11 possesses about the same gravity as No. 1 (bottled lager). For this type of beer it will be noticed that, and especially in comparison with the lager, the maltose-dextrin ratio is high. The ash is very low, and the nitrogen also is very low. This beer (No. 11) is probably chilled and carbonated, hence the low nitrogen proportion. The flavour in this, and in other cases where chilled beers are compared with the naturally bottled matured articles, is not particularly good. In the case of another light bottled beer, namely No. 12, it is seen that the attenuation is rather low, and that there is a great excess of maltose. This excess of maltose in bottled beers is perhaps somewhat to be accounted for by the use of priming to assist in bringing to rapid condition.

It is interesting to note the wide variation in the gravity of the London mild ales, ranging as it does between 1040.9 to 1056.8. There is also considerable variation in quality, as might have been expected.

Of all the light beers Lager shows the lowest alcohol content and the greatest proportion of solids compared with alcohol. It will be noticed also that in the case of the dark lager particularly, the nitrogen is high, showing that peptonisation has proceeded further in this class of beer than in that of ordinary English brewing. This, of course, is a well-known fact. It will be also noticed that the proportion of dextrin to maltose is much higher in the lager beer than in most of the other types. In fact only in two cases in the British beers is the amount of dextrin more than in the maltose, namely in the case of two Burton beers, Nos. 9 and 10.

There are interesting differences between the Burton and London draught beers. Generally speaking, Burton beers are lighter in colour, are of higher quality, of higher gravity, and more highly hopped than the London beers. It may be generally assumed also that they are made from finer materials. Compare, for instance, a London and a Burton draught beer of similar gravity—for instance, No. 9 with No. 5 or No. 7—we find the Burton beer is more highly attenuated, and that it contains relatively more dextrin, and that the character generally is that of a more stable beer. This is largely due to the method of brewing and also to the greater amount of hops. Comparing the bottled beers, there is among them no London beer that can be compared with the famous Burton pale ales.

2. *Stout.*—The first point that strikes one in regard to the stouts is their very heavy gravity. This heavy gravity means, of course, very large amounts of total solids and a considerable amount of alcohol; the solids to alcohol ratio is, however, higher than in the case of the lager types. There are interesting differences again between the Dublin and London types of black beers. The London beers are full and sweet and contain a large amount of maltose, whereas the Irish beers are more dextrinous and much drier to the taste. It is observable that the nitrogen and phosphorus contents of these beers are also very high.

With regard to Lager it may be said, comparing the lager beers with the other low-gravity cheap beers, that, although one cannot account for individual taste, there is no doubt that the lager beer is decidedly superior from most points of view. It is elegant, light and bright, nutritive, and of low alcoholic content. The low-gravity draught London ales can certainly not be described as elegant or superior from a gastronomic point of view. It may be fairly said, therefore, with regard to low-gravity light beverages that lager is a superior article.

As we explained elsewhere lager beer is an exceedingly fine product, requiring extreme care both in its production and, further, in its preservation. The import into this country of German lager beer is only rendered possible by, to some extent, sacrificing one of its most valuable characteristics, namely its lightness, and by the frequent use of salicylic acid as a preservative. A lager beer produced locally, and therefore independent of the exigencies of transit, is a distinct desideratum and possessed of obvious advantages over the imported product.

### PART II.—THE PHYSIOLOGICAL, DIETETIC, AND MEDICINAL PROPERTIES OF BEER.

### The Physiology of Thirst.

Having discussed at some length what beer is and how it comes to be what it is, we shall conclude our report by considering what are the dietetic and medicinal uses of beer. A mere glance at our analytical tables will make it at once obvious that beer, unlike tea, mineral waters and many other beverages, is not a pure stimulant nor a mere thirst quencher, but actually a food, and capable of supplying to the human body assimilable material of definite calorimetric or energy value. To speak first of perhaps the least important properties of beer, we may consider its value as a thirst quencher.

*The physiology of thirst* is, perhaps, a little more complicated than would appear on the surface. The most essential physiological basis of thirst, which, by the way, is one of the most distressing of human sufferings, far more distressing than hunger, as we know from the records of fasting experiments, is an actual want on the part of the body of fluid. By sweating (usually under muscular exercise), by excessive secretion of urine, by diarrhœa, or by any other means which has occasioned a loss of moisture out of proportion to in-take, thirst, or in other words the irresistible inclination to drink, may be produced. Thirst of this kind is invariably associated with the sensation of dryness about the mouth, the pharynx, and the naso-pharynx, due essentially to the fact that, owing to the diminished quantity of water available for the wants of the body, the secretion of saliva, the natural moistening agency of the above parts, is much diminished.

### Artificial Thirst.

In contra-distinction to the above true physiological thirst, there exists nowadays what we may term a spurious thirst, or the sensation of thirst without the actual need on the part of the body for moisture. This thirst practically depends entirely upon dryness of the pharynx, the palate, the naso-pharynx and adjoining parts, and is in many cases due to a deficiency in quantity or quality of the saliva. This phenomenon often depends upon the vagaries of modern diet and habits, chief amongst which may be mentioned, first and foremost, cigarette smoking, the taste for highly spiced food, which is now fast spreading even to the fair sex, and last, but not least, the taste for mineral waters, and especially those supersaturated artificially with carbonic-acid gas. The products of the combustion of paper, the spices and salted almonds, the bubbles of carbonic-acid gas evolved from mineral waters, in their course down the swallowing tract all tickle for the moment our palates and we like them, but just as surely as dropping water wears away the stone, so do these constant irritants destroy the secreting glands of those parts exposed to them and produce the dry-throated, husky or nasal voiced, and spuriously thirsty individual. Breathing through the mouth instead of through the nose by causing a constant current of drying air through parts which should be devoted to taste, tends still further to make the mouth and throat dry and increase thirst.

### Thirst Quenching.

Whatever may be the kind of thirst from which we suffer, to assuage it fully we must get and keep the palate, the pharynx, and the naso-pharynx moist. We can best do this by simultaneously producing two phenomena. Firstly, by choosing a drink which will cover the above parts with a gummy or mucilaginous film, and thus prevent them from losing to the air what moisture they possess; and, secondly, by choosing a drink of such a taste as will in the course of swallowing reflexly stimulate the secretion of saliva. Either of these agencies will separately quench thirst, but they will do so more completely when they can act together.

Sugary and gummy solutions to some extent quench thirst by means of protecting the mucous membrane from drying, but they are not really good thirst quenchers because they have no reflex stimulating effect upon the secretion of saliva. Beer and some of the bitter mucilaginous infusions have the same mechanical effect in keeping the mucous membrane moist as the gums and the dilute syrups, but in addition to this, by virtue of their bitter taste, the former stimulate reflexly the secretion of saliva, and thus cause a flow of the secretion specially designed to keep the mouth and throat moist. To obtain the maximum thirst-quenching effect from beer it should not be swallowed too quickly, and a beer should be chosen possessing a relatively large quantity of total solids, and especially of dextrine, and also possessing a clean bitter taste. Thin, highly effervescent ales should be avoided as they are spurious thirst quenchers, the rapidly evolved bubbles of carbonic-acid gas cooling the pharynx and palate for the moment, but subsequently irritating it and making it drier than before. One of the best thirst quenchers is good lager beer. This is light in alcohol, but strong in taste and rich in dextrine.

### Effect of Beer upon Digestion.

Beer, if taken alone, is rapidly absorbed from the stomach. Fairly exact experiments have shown that half a pint of beer when taken by itself leaves the stomach in little over an hour, in fact practically as rapidly as water. From this we may assume that a glass of good ale between meals is an easy method of supplying energy to the body.

Beer when taken with meals appears slightly to retard gastric digestion, so far as experiments *in vitro* are concerned. In the living individual, however, this retarding influence appears hardly to exist, and quantities of beer up to a pint are practically without influence. Indeed it is probable that bitter ale taken with meals, by virtue of the stimulant action it exerts upon the secretion of gastric juice, actually promotes gastric digestion. Here, again, the kind of beer taken is by no means without influence. We should at once preclude the use of artificially highly carbonated bottled beer, and suggest that the best beer is one with a high extract value in proportion to its alcohol, and also one in which the dextrine is equal, or approximately equal, to the maltose. In certain patients beer when taken with meals tends to produce flatulence. We are quite sure in many of these instances the patient or his doctor has not discriminated sufficiently between the different kinds of beer. Beer, however, is not likely to suit patients suffering from fermentative dyspepsia or patients suffering, as the above patients generally do, from considerable gastric dilatation, as it obviously contains a considerable quantity of fermentable material which, if it remains for long in a dilated stomach already richly infected with stray fermentative organisms, must undergo fermentation, which will result in the production of much carbon-dioxide gas and acids such as acetic. If, however, in these patients, who are frequently emaciated, special means are adopted to get in the first place a diminished activity of these fermentative organisms by the use of suitable antiseptics, and in the second place a rapid emptying of the stomach by means of position and massage, beer, and especially stout, may be used with advantage as a tonic and nourishing agent. In simple hyper-chlorhydria, or that form of dyspepsia characterised by an increase in the proportion of hydrochloric acid in the gastric juice and accompanied by pain, most marked an hour before meals, and not associated with dilatation of the stomach, good beer and stout may be used as food and, indeed, as remedial agents. Their gummy content soothes the irritated gastric mucous membrane, and their acidity, due as it is mostly to acid phosphates, is practically without influence.

### Therapeutic Uses of Beers.

In prescribing beer or stout we must never forget that we are prescribing foods, part of the food value of which is due to alcohol and part to carbo-hydrate material, and that in this form both these food-stuffs are diluted with a large quantity of water, and hence considering their food value are relatively bulky. From this it follows that we must not prescribe beer or stout for any patient in whom either alcohol, carbo-hydrate, or a relatively large quantity of liquid are contra-indicated.

It is unnecessary here to specify individual diseases, but, speaking generally, we may say that beer and stout are not suitable foods for the obese, for the subjects of renal disease or glycosuria or diabetes. Before leaving this medicinal aspect of the subject, a special use of beer and stout, and especially the latter, must find mention. We refer to their hypnotic action. Stout is one of the most harmless and best hypnotics we possess, and is often far more efficacious in the treatment of insomnia than drugs. Precisely how these substances act we do not know, but part of their action is certainly due to the hypnotic principle contained in hops, and the more heavily these beverages are hopped the more marked is their hypnotic action. An average dose for the purpose is half a pint, and this should be taken last thing at night, as long after an early dinner or supper as possible.

We have said so much in what has gone before concerning the food value of beer and stout, both from the chemical and physiological standpoint, that we need add but little here. Beer and stout must be regarded as the most nourishing alcoholic beverages we possess, and whatever may be said concerning the value of alcohol as a food, there can be no question of the food value of beer and stout. They contain all the elements of a typical diet with the exception of fat, and in a proportion approximately physiological. If we are content to measure the worth of a food by its calorimetric value, the fact is that a glass of good ale is approximately as nourishing as a glass of milk, and that a quart of good beer is equivalent to nearly a quarter of a pound of bread. Amongst beers good lager beer stands in a special position, because from the alcoholic standpoint it is light and from the nutritive standpoint it is heavy. It has been approximately estimated that the number of calories or energy equivalents required by an ordinary working-man *per diem* are three thousand; a quart of good beer, quite apart from its alcoholic content, contains in itself one-sixth of the total energy required daily.

### Final Considerations.

In the foregoing pages we have endeavoured to place the medical man as concisely as possible in possession of the facts concerning the manufacture, dietetic properties and medicinal uses of beer and stout, and have also, we hope, enabled him to discriminate between the bad, the good, and the indifferent. All flesh is not the same flesh, equally surely all beer is not the same beer; and just as the medical man often has occasion to prescribe one kind of proteid food for one patient, another kind of proteid for another, so to get the true effect of malt liquors must he be able to discriminate between them. We feel sure that many of the evil dietetic effects which have followed the use of these beverages have been due to the consumer having been supplied with beer or stouts of an indifferent character. Beer and stout of proper quality and given in proper dose, and used in suitable cases, will be found amongst the most useful dietetic and indeed medicinal agencies we possess.

## Figures and Tables

**Figure f1:**
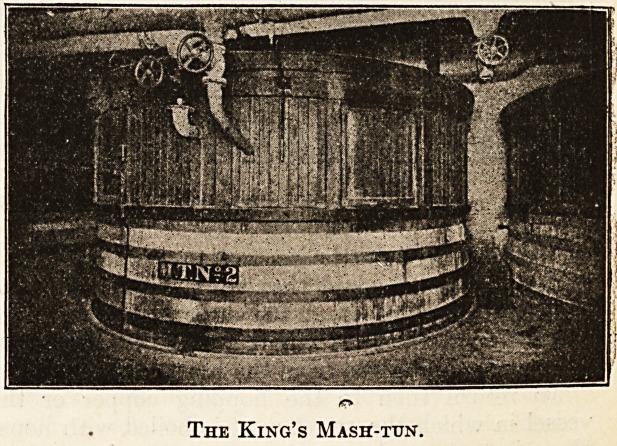


**Figure f2:**
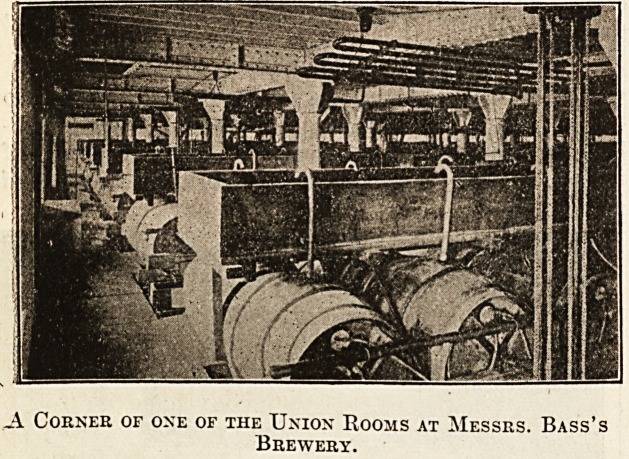


**Figure f3:**
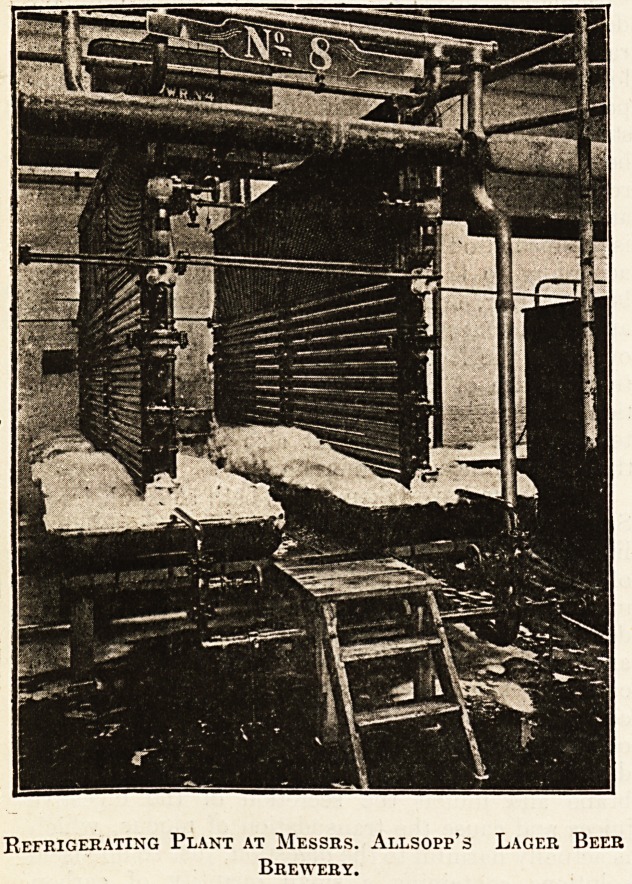


**Figure f4:**